# Detection of leptospiral antibodies and DNA in freshwater fish

**DOI:** 10.3389/fvets.2025.1663896

**Published:** 2025-09-01

**Authors:** LaRoy Brandt, Emily Willems, Joey Morgan, Lauren Wisnieski, Sloane Boukobza, Allison Geer, Conner Duke, Scott Brovarney, Brooke Noah, Michele D. Coarsey, Hemant Naikare, Shashi S. Verma, Agnes Mary Vanderpool, Ashutosh Verma

**Affiliations:** ^1^School of Mathematics, Sciences and Health Professions, Harrogate, TN, United States; ^2^Cumberland Mountain Research Center, Harrogate, TN, United States; ^3^College of Veterinary Medicine, Lincoln Memorial University, Harrogate, TN, United States; ^4^Tifton Veterinary Diagnostic and Investigational Lab, College of Veterinary Medicine, University of Georgia, Tifton, GA, United States; ^5^J. Frank White Academy, Harrogate, TN, United States; ^6^Center for Infectious, Zoonotic and Vector-Borne Diseases, Lincoln Memorial University, Harrogate, TN, United States

**Keywords:** *Leptospira* spp., fish, Powell River, Appalachia, Cumberland Gap Region

## Abstract

Leptospirosis is an important zoonotic disease that is maintained in populations due to chronic kidney infection of reservoir mammals. Previous work from our lab has identified rodents, voles, shrews, chipmunks and several species of amphibians and reptiles as hosts of *Leptospira* spp. in the Cumberland Gap Region of Kentucky, Tennessee, and Virginia. The aim of this study was to determine if fish contribute to the maintenance of the pathogen in the aquatic environment. Fish (*n* = 238), belonging to 19 genera, were collected from seven different locations in the Powell River in East Tennessee. Fish kidneys were harvested and screened for leptospiral DNA using a TaqMan quantitative polymerase chain reaction (qPCR) assay that targets pathogenic *Leptospira* spp. Blood samples were collected for measuring leptospiral antibodies using microscopic agglutination test (MAT). Of the 238 fish screened, 11 were positive by either qPCR or MAT (4.62%; 95% CI: 2.33–8.12). Of these 3 (3/238; 1.26%; 95% CI: 0.26–3.64) were positive by qPCR and 8 (8/237; 3.38%; 95% CI: 1.47–6.54) were found to have antibodies to at least one leptospiral serovar by MAT. This is the first report of leptospiral DNA detection in fish kidneys, providing insights on the potential role of fish in the epidemiology of leptospirosis in the region.

## 1 Introduction

Leptospirosis is a water-borne zoonotic disease primarily caused by the pathogenic species of *Leptospira*. The disease in humans is characterized by protean manifestations with clinical signs ranging from flu-like symptoms in most cases to life-threatening multisystemic organ failure in a smaller subset of patients. In animals, leptospirosis leads to significant production losses through decreased milk production, infertility, spontaneous abortion, hepatorenal failure, and death (1, 2). The etiological agent lives in the proximal renal tubules of chronically infected animals and is shed in their urine, thus contaminating surface water, soil, streams, ponds, and rivers. The infection is acquired by other animals, and humans when they come in direct contact with urine from infected animals or water or soil contaminated by such urine (3–5). Pathogenic *Leptospira* enter hosts through small cuts on the skin, or intact mucus membranes.

Although rodents are the primary reservoir host of *Leptospira*, many species of small mammals have been known to carry the pathogen (6). Historically, mammals have been the primary host species maintaining leptospires in the environment; however, several studies have shown that various species of reptiles and amphibians can also carry this pathogen in their kidneys (7–14). These host species hold an ecological niche both on land and in water, thus potentially expanding the reach of this pathogen to aquatic life.

Previous studies from our lab suggest that *Leptospira* is enzootic in the Cumberland Gap Region (CGR) of South-Central Appalachia, continuously circulating among small wild mammals, herpetofauna, livestock and shelter dogs (14–16). One of those studies also provided evidence of leptospiral contamination in environmental water in the region (16). Since no information from the United States is available on *Leptospira* infection in fish, we conducted this study to investigate the association by testing freshwater fish for the presence of pathogenic *Leptospira* and leptospiral antibodies. Kidneys of fish were screened for the presence of leptospiral DNA using quantitative polymerase chain reaction (qPCR), and leptospiral antibodies were measured using microscopic agglutination test, the gold standard in leptospiral serology.

## 2 Materials and methods

### 2.1 Ethics statement

All fish capture protocols were based on the Guidelines for the Use of Fishes in Research established by the American Fisheries Society (17) and were reviewed and approved by the Animal Care and Use Committee at the Lincoln Memorial University (IACUC # 2102-RES). Fish in this study were collected under an aquatic collection permit issued through Tennessee Wildlife Resources Agency.

### 2.2 Study area and sample collection

**Figure 1:**
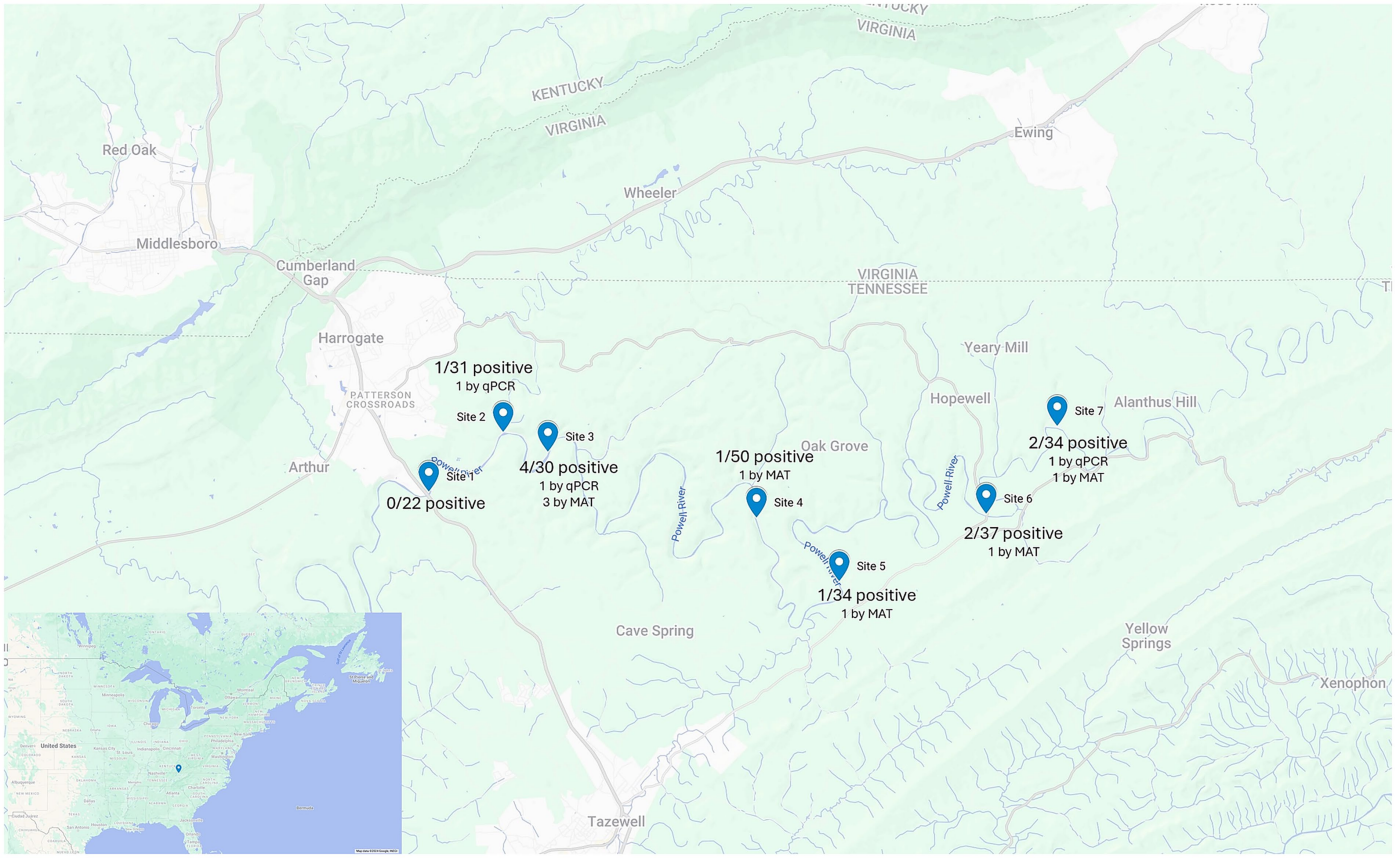
Map showing the seven fish collection sites along the Powell River in Tennessee, including the number of fish collected at each site, the number of positive fish, and the diagnostic methods used. Map created with Google My Maps.

The sampling area included seven different sites (Sites 1–7) within a 47-mile segment of the Powell River in Claiborne County, Tennessee, extending from River Mile 112 to 65 (Figure 1). From each of the seven sites, no more than five fish per species were collected and the total number of fish collected across all species did not exceed 50 (Table 1). Only common species of fish within the Powell River were collected.

**Table 1:** Number of fish collected at different collection sites in Powell River, Tennessee, USA.

Collection site	Number of fish collected	Fish positive by qPCR	Fish positive by MAT	Total positive
Site 1	22	0	0	0
Site 2	31	1	0	1
Site 3	30	1	3	4
Site 4	50	0	1	1
Site 5	34	0	1	1
Site 6	37	0	2	2
Site 7	34	1	1	2

A total of 238 fish of 19 genera were collected from seven different locations along the Powell River in Tennessee. Fish were collected in-stream with the use of a Smith-Root electrofishing backpack unit, and as described elsewhere (18–23). Electrofishing introduces an electric current into the water to momentarily stun fish to allow for collection by hand-net. Once collected by hand-net, fish were identified to species using dichotomous keys provided in (24). Fish collected for research were euthanized by exposure to lethal levels of the sedative MS-222, tricaine methane sulfonate. Once the animals were anesthetized and humanely euthanized, blood samples were collected from the tail vein, transported to lab on ice, centrifuged and stored at –20 °C until tested for MAT. The fish were also transported to the laboratory on ice, where the renal tissue was harvested and stored at –20 °C until further processing.

### 2.3 DNA extraction and quantitative polymerase chain reaction (qPCR)

Renal tissue from one or both kidneys was pooled and processed for DNA extraction using the DNeasy Blood and Tissue Kit (Qiagen, Valencia, CA, USA), following the manufacturer’s instructions. Leptospiral DNA in fish renal tissue was detected using a TaqMan based qPCR that targets a 242 bp region of leptospiral *lipl32* gene as described by Stoddard et al. (25). The assay was performed in a MicroAmp Fast Optical 96-well reaction plate (Applied Biosystems, Foster City, CA, USA) using QuantStudio 3 and Platinum Quantiative PCR SuperMix-UDG (Invitrogen, Carlsbad, CA, USA). Each reaction was performed in a 25 μL final volume, using 500 nM of LipL32-45F (forward primer; 5’-AAGCATTACCGCTTGTGGTG-3′), 500 nM of LipL32-286R (reverse primer; 5’-GAACTCCCATTTCAGCGATT-3′) and 100 nM of LipL32-189P (probe; FAM-5’-AAAGCCAGGACAAGCGCCG-3’-BHQ1) (25). The standard curve was generated with leptospiral DNA equivalent to 10^7^–10^0^ genome units. Each column on the assay plate had a no-template control. Thermal conditions used were as follows: a holding stage of 95 °C for 20 s, and 40 cycles of 95 °C for 3 s and 60 °C for 30 s. Each test DNA sample was tested in duplicate and repeated twice or more.

### 2.4 Microscopic agglutination test (MAT)

A total of 237 fish sera were screened for the presence of leptospiral antibodies by Microscopic Agglutination Test (MAT). The MAT was performed following the standardized protocol recommended by the World Organization for Animal Health (26). Briefly, two-fold serum dilutions from 1:100 to 1:6,400 were tested against leptospiral serovars Pomona strain Pomona (serogroup Pomona), Hardjo type Prajitno strain Hardjoprajitno (serogroup Sejroe), Grippotyphosa strain Andaman (serogroup Grippotyphosa), Icterohaemorrhagiae strain M20 (serogroup Icterohaemorrhagiae), Canicola strain Hond Utrecht IV (serogroup Canicola), Bratislava strain Jez Bratislava (serogroup Australis), and Autumnalis strain Akiyami A (serogroup Autumnalis) (National Veterinary Services Laboratories, Ames, Iowa, IA, USA). MAT titer was defined as the reciprocal of the highest dilution of a serum sample that agglutinated more than half of leptospires. Titers of more than or equal to 1:100 were considered positive for the presence of leptospiral antibodies.

### 2.5 Statistical analysis

The ‘cii” command in STATA version 17.0 (College Station, TX) was used to calculate 95% confidence intervals for the prevalence estimates.

## 3 Results

The collected fish included 40 Sunfish (*Lepomis*), 34 Rockbass (*Ambloplites*), 34 Chubs (*Erimystax*, *Nacomis*, *Semotilus*, *Hybopsis*), 30 Stonerollers (*Campostoma*), 24 Shiners (*Cyprinella*, *Notemigonus*, *Luxilus*), 22 Hogsuckers (*Hypentelium*), 16 Bass (*Micropterus*), 13 Shortnosed Redhorse (*Moxostoma*), 12 Common Log Perch (*Percina caprodes*), 6 Darters (*Percina aurantiaca*, *Etheostoma*), 5 Longnose Gar (*Lepisosteus*), 1 Catfish (*Pylodictus*), and 1 Bluntnose Minnow (*Pimephales*).

Of the 238 fish kidneys screened, leptospiral DNA was detected in three fish (3/238; 1.26%; 95% CI: 0.26–3.64) (Table 2). The qPCR-positive fish included two Green Sunfish (*Lepomis cynellis*) and one Rockbass (*Ambloplites rupestris*). Leptospiral concentration in fish kidneys ranged from 42–1.5×10^3^ genomic equivalents (GE)/gram of renal tissue, with an average concentration of 626 GE/gram of renal tissue. Although two of the three positive fish were Green Sunfish, the data are insufficient to determine species-specific predisposition to leptospiral carriage. Additionally, due to the small kidney size in some fish species, renal tissues from both kidneys were pooled for DNA extraction. As a result, the current data cannot differentiate between unilateral and bilateral kidney infections.

**Table 2:** Leptospiral carriage and antibodies in freshwater fish in Powell River, Tennessee.

Sample type	Positive	Number of samples	Percent positive	95% confidence interval	Test
Kidney tissue	3	238	1.26%%	0.26–3.64%	qPCR
Sera	8	237	3.38%	1.47–6.54%	MAT
Total (kidney and sera)	11	238	4.62%	2.33–8.12%	qPCR and MAT

Of the 237 fish sera tested, 8 (3.38%; 95% CI: 1.47–6.54) had antibodies to at least one leptospiral serovar (Table 2). The majority of positive fish (*n* = 7) reacted with one serovar., with 50 % of the MAT-positive fish (3 Rockbass and 1 Sunfish) reactive to serovar Icterohaemorrhagiae (Table 3). While two serum samples belonging to a Smallmouth Bass (*Micropterus dolomieu*) and a River Chub (*Nacomis micropogon*) reacted with serovar Autumnalis, one fish (Sunfish) contained antibodies to serovar Grippotyphosa. A Shortnosed Redhorse (*Moxostoma macrolepidotum*) reacted with serovars Pomona, Hardjo, and Grippotyphosa. All positive sera had titers greater than or equal to 1:100, except for one sera that had a titer greater than or equal to 1:200 for serovar Autumnalis (Table 3). None of the screened fish sera contained antibodies to serovars Canicola or Bratislava. No tested fish had both leptospiral antibodies and leptospiral DNA.

**Table 3:** Serovar reactivity of microscopic agglutination test (MAT)-positive fish.

Species name	Common name	Pom*	Har*	Grippo*	Ictero*	Cani*	Bratis*	Autum*
*Ambloplites rupestris*	Rockbass	N^#^	N	N	**1:100**	N	N	N
*Ambloplites rupestris*	Rockbass	N	N	N	**1:100**	N	N	N
*Lepomis cyanellis*	Green Sunfish	N	N	N	**1:100**	N	N	N
*Ambloplites rupestris*	Rockbass	N	N	N	**1:100**	N	N	N
*Lepomis megalotis*	Longear Sunfish	N	N	**1:100**	N	N	N	N
*Moxostoma macrolepidotum*	Shortnosed Redhorse	**1:100**	**1:100**	**1:100**	N	N	N	N
*Micropterus dolomieu*	Smallmouth Bass	N	N	N	N	N	N	**1:200**
*Nacomis micropogon*	River Chub	N	N	N	N	N	N	**1:100**

Pom, Pomona; Har, Hardjo; Grippo, Grippotyphosa; Ictero, Icterohaemorrhagiae; Cani, Canicola; Bratis, Bratislava; Autum, Autumnalis. #N=Negative. Values in bold indicate MAT-titer.

In total, 11 fish (11/238; 4.62%; 95% CI: 2.33–8.12) had either leptospiral DNA present in their kidneys or serum antibodies reactive to at least one leptospiral serovar tested. Six of the seven collection sites (Figure 1) had at least one fish positive for the presence of leptospiral antibodies or leptospiral DNA. The highest number of fish testing positive for leptospiral DNA or antibodies was observed at Site 3 (*n* = 4), followed by Sites 6 and 7 (*n* = 2) and Sites 2, 4, 5 (*n* = 1). No fish collected from Site 1 tested positive for either test (Table 1).

## 4 Discussion

Previous studies from our laboratory have shown in the Cumberland Gap Region of South-Central Appalachia, a variety of small wild mammals (rodents, shrews, voles, cottontails and chipmunks) as well as several species of amphibians and reptiles (Green frog, American bullfrog, American toad, Northern slimy salamander, Eastern newt, Common snapping turtle, Garter snake, Northern water snake, and Ringneck snake) can carry *Leptospira* spp. in their kidneys. Since many species of amphibians that tested positive in our previous study live in or around water bodies, we investigated if fish too contribute to maintenance of leptospires in the aquatic environment.

Our study provides evidence of leptospiral presence in common freshwater fish species found in the Powell River. Leptospiral DNA was detected in the kidneys of Green Sunfish and Rockbass. Additionally, leptospiral antibodies were found in the sera of Rockbass, Sunfish, Smallmouth Bass, River Chub, and Shortnosed Redhorse. In our knowledge, there are only two published reports that directly investigated *Leptospira* in fish (27, 28), although a few studies have examined the infection as an occupational hazard associated with fish farming. (29–31). In a 2014 study from Tanzania, 26 of 48 (54.2%) tested Catfish, Tilapia and Eel fish were positive when tested serologically against locally prevalent serovars Sokoine, Kenya, Pomona, and Hebdomadis (28). The high prevalence in fish in that study (28) could be due to small sample size, overall high prevalence of the disease across multiple reservoir species in that region, or low MAT cut-off values. Although the seroprevalence observed in our study was lower than that reported in the study by Mgode et al., our study provides the first documented evidence of leptospiral renal carriage in fish. These findings suggest that fish may play a role in the maintenance of pathogenic *Leptospira* spp. within aquatic ecosystems. Moreover, the detection of leptospiral DNA in the kidneys of freshwater fish underscores the importance of proper food safety measures. Thorough cooking and, in case of raw fish dishes, freezing at sufficiently low temperatures are critical to reduce exposure to this zoonotic pathogen.

The Powell River, a 195-mile-long body of water spanning from the US states of Virginia to Tennessee, is named as the second most diverse aquatic system in the nation by the Environmental Protection Agency and serves as a highly populated crossroad for wildlife and humans (32). The 47-mile-long stretch of the river sampled in this study runs through Claibourne County, Tennessee. It is notable for its high foot-traffic as community members kayak, fish, and swim in the river throughout late summer and early fall. In addition to aquatic activities, many bring household pets to walk or swim in the area. The Powell River is also home to highly diverse fauna, and acts as an overlapping point of community, domesticated animals, and wildlife. The wildlife in this area, which includes deer, foxes, and small mammals, rely upon this body of water for survival. The overlapping habitats of various potential hosts, and use of this environment by humans for recreational activities, may facilitate the continuous circulation of this pathogen among terrestrial, amphibian, and aquatic species.

The seven collection sites across the Powell River in Tennessee maintained varying degrees of flowing water, with portions being still and the river itself being home to a variety of fish species. The collected nineteen genera of fish included both bottom-dwelling fish as well as species that spend a majority of their time at the surface of the water. Although this study provided evidence for renal carriage of *Leptospira* spp. in fish, there are still many unknown aspects of this host-pathogen relationship. For example, how do structural and physiological differences in fish kidneys impact their role in leptospiral maintenance and shedding? Do other organs in fish harbor leptospires? Additionally, there is a lack of data on the prevalent serovars in fish. We screened fish sera against the most prevalent leptospiral serovars in the US, but there is a possibility that other serovars are more prevalent in aquatic species and there may also be regional variations. All or some of these factors may have a role in relatively low leptospiral prevalence in fish in this study.

We did not attempt leptospiral culture, nor did we include intermediate leptospiral strains in the MAT panel or used a molecular assay that can detect intermediate strains. These limitations, along with the absence of genotyping of *Leptospira* in qPCR-positive kidney samples, should be addressed in future studies to further characterize the diversity and potential pathogenicity of leptospiral strain in fish.

In summary, this work confirms the presence of leptospiral antibodies and DNA in freshwater fish, adding to the growing body of evidence that non-mammalian species may play a role in the ecology of leptospirosis. Whether fish contribute to the transmission cycle or act as a mere bystander of infection remains to be investigated.

## Data Availability

The original contributions presented in the study are included in the article/supplementary material, further inquiries can be directed to the corresponding author.
